# Therapy related acute myeloid leukemia with blast cannibalism and cytophagocytosis

**DOI:** 10.1002/jha2.49

**Published:** 2020-06-28

**Authors:** Habib Moshref Razavi, Thomas Covello

**Affiliations:** ^1^ Division of Hematopathology and Transfusion Medicine Fraser Health Authority Vancouver British Columbia Canada; ^2^ Department of Pathology and Laboratory Medicine University of British Columbia Vancouver British Columbia Canada

**Keywords:** AML, cannibalism, cytophagocytosis, dyserythropoiesis

## Abstract

The case of an 83‐year old male patient is described. He presented to our hospital with a 2 week history of intermittent syncope and was diagnosed with atrial fibrillation. His CBC showed bicytopenia with anemia and neutropenia in context of a leucoerythroblastic blood film and blastemia. As the patient was previously diagnosed with stage IV diffuse large B cell lymphoma; treated with CHOP‐R a bone marrow biopsy was requested to rule out acute leukemia. A cellular bone marrow aspirate showed presence of a myeloblast infiltrate (32.6%; confirmed by flow cytometry). Interestingly blast phagocytosis of terminally differentiated cells along with cannibalism of other blasts was identified. The diagnosis of therapy‐related acute myeloid leukemia was reached and a palliative consultation was requested. To our knowledge, this is the first report of blast cytophagocytosis and cannibalism associated with a therapy‐related acute myeloid leukemia with a complex karyotype.

An 83‐year‐old male patient with severe anemia and thrombocytopenia is described. The patient has a prior history of stage IV diffuse large B‐cell lymphoma diagnosed in 2015 and was previously treated with Cyclophosphamide, Doxorubicin Hydrochloride, Vincristine (Oncovin), Prednisone, and Rituximab (CHOP‐R). He presented to our hospital with a 2‐week history of intermittent syncope and was diagnosed with atrial fibrillation. A full blood count (FBC) showed a bicytopenia with a hemoglobin of 73 g/L and platelet count at 28 × 10^9^/L. Neutrophil count was increased and he had circulating blasts (7.9 × 10^9^/L). His peripheral blood film showed a leucoerythroblastic picture, dysplastic nucleated red cells, and circulating blasts (not shown). The bone marrow aspirate (image, May‐Grünwald‐ Giemsa) showed a large blast infiltrate (32.6%). These were moderately sized cells with a high N/C ratio, showing scant amount of blue/grey cytoplasms without Auer rods. Occasional cytoplasmic blebbing and abundant cytoplasmic fragmentation was present. These cells also possessed round nuclear contours with vesicular chromatin, and occasional large nucleoli. Interestingly blast cytophagocytosis was seen. Specifically blast ingestion of erythrocytes, orthochromic normoblasts (top row), lymphocytes such as large granular lymphocytes (LGLs), granulocytes, and other blasts (middle panel) was identified. A trephine biopsy showed a hypercellular bone marrow with a large blast infiltrate (Hematoxylin and Eosin (H&E) ×10 bottom row) that was partially Cluster of Differentiation (CD) 34^+^. CD117 and e‐cadherin showed a larger subset of positively stained blasts throughout (not shown). Black arrows highlight blast cytophagocytosis in situ (×50 H&E and CD117 stain, respectively). Aspirate flow cytometric immunophenotyping identified a blast infiltrate expressing CD34, weak CD45, CD117, human leucocyte antigen‐DR isotype (HLADR), weak CD13, CD33, weak CD4, and weak myeloperoxidase (cMPO, 9% of nucleated cells). Cytogenetic studies showed a complex karyotype: 43,X,‐Y,add(2)(p23),‐7,‐9,‐9,add(14)(p11.1),‐15,‐17,‐19,‐21,‐21,+mar1,+mar2,+mar3,+mar4,+mar5,+mar6[7]/45,X,‐Y[4]. With a large blast infiltrate and significant dyserythropoiesis along with a previous history of CHOP‐R therapy diagnosis of therapy‐related acute myeloid leukemia was reached. The patient was admitted and a palliative consultation was requested.

The phenomenon of blast cytophagocytosis has previously been reported and is usually associated with myelomonocytic or monocytic blast morphology. Less often erythroblasts and megakaryoblasts have also been implicated. There are several reports of recurrent cytogenetic abnormalities associated with blast cytophagocytosis including t(8;16)(p11;p13), inv(8)(p11q13) and t(16;21)(p11;q22) among others. Although cytophagocytosis included many terminally differentiated cells, blast ingestion and cannibalism by other blasts were also seen. Blast cytophagocytosis in general and blast cannibalism in particular is a rare event in acute myeloid leukemia.

**FIGURE 1:**
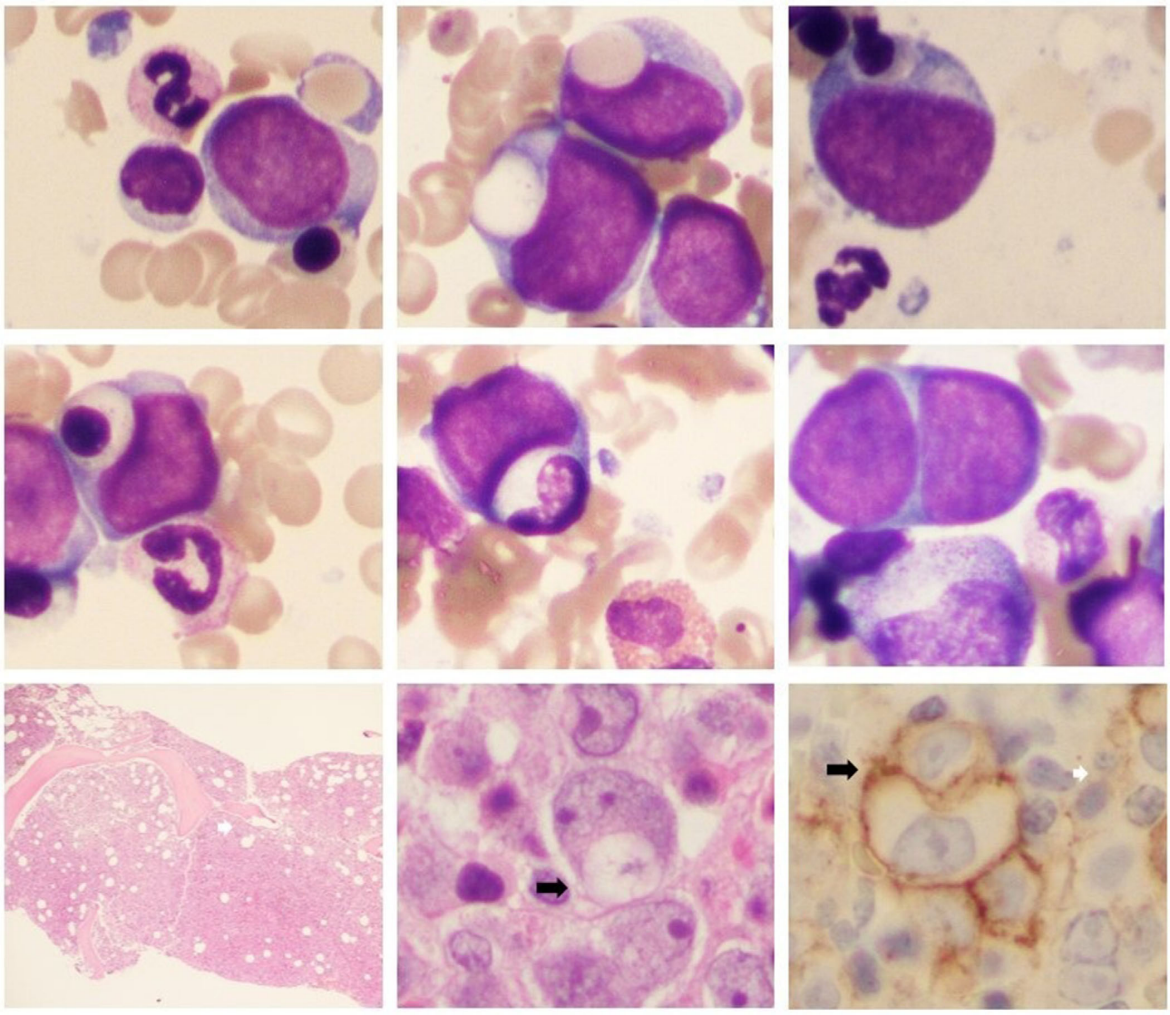
Blast phagocytosis is shown in the bone marrow aspirate (top two rows) as well as in situ (lower panel). Please see text for details.

